# Determining the Predictors of Recurrence or Regrowth Following Spinal Astrocytoma Resection: A Systematic Review and Meta-Analysis

**DOI:** 10.3390/brainsci14121226

**Published:** 2024-12-04

**Authors:** Harry Hoang, Amine Mellal, Milad Dulloo, Ryan T. Nguyen, Neil Nazar Al-Saidi, Hamzah Magableh, Alexis Cailleteau, Abdul Karim Ghaith, Victor Gabriel El-Hajj, Adrian Elmi-Terander

**Affiliations:** 1 Faculty of Medicine, Department of Clinical Neurosciences, University of Geneva, 1205 Geneva, Switzerland; harry.hoang@hug.ch (H.H.); alexis.cailleteau@gmail.com (A.C.); 2 Department of Neurosurgery, Lausanne University Hospital and University of Lausanne, 1011 Lausanne, Switzerland; 3 Faculty of Biology and Medicine, University of Lausanne, 1011 Lausanne, Switzerland; milad.dulloo@gmail.com; 4 Mayo Clinic Neuro-Informatics Laboratory, Mayo Clinic, Rochester, MN 55902, USA; nguyenry@hawaii.edu (R.T.N.); neilalsaidi@gmail.com (N.N.A.-S.); hmagableh@alfaisal.edu (H.M.); abdulkarimghaith@gmail.com (A.K.G.);; 5 Department of Clinical Neuroscience, Karolinska Institutet, 171 77 Stockholm, Sweden

**Keywords:** spinal astrocytoma, recurrence, predictors, mortality, prognosis, adjuvant radiotherapy

## Abstract

Background/Objectives: Spinal astrocytomas (SA) represent 30–40% of all intramedullary spinal cord tumors (IMSCTs) and present significant clinical challenges due to their aggressive behavior and potential for recurrence. We aimed to pool the evidence on SA and investigate predictors of regrowth or recurrence after surgical resection. Methods: A systematic review and meta-analysis were conducted on peer-reviewed human studies from several databases covering the field of SA. Data were collected including sex, age, tumor location, extent of resection, histopathological diagnosis, and adjuvant therapy to identify predictors of SA recurrence. Recurrence was defined as failure of local tumor control or regrowth after treatment. Results: A total of 53 studies with 1365 patients were included in the meta-analysis. A postoperative deterioration in neurological outcomes, as assessed by the modified McCormick scale, was noted in most of the patients. The overall recurrence rate amounted to 41%. On meta-analysis, high-grade WHO tumors were associated with higher odds of recurrence (OR = 2.65; 95% CI: 1.87, 3.76; *p* = 0.001). Similarly, GTR was associated with lower odds of recurrence compared to STR (OR = 0.33; 95% CI: 0.18, 0.60; *p* = 0.0003). Sex (*p* = 0.5848) and tumor location (*p* = 0.3693) did not show any significant differences in the odds of recurrence. Intraoperative neurophysiological monitoring was described in 8 studies and adjuvant radiotherapy in 41 studies. Conclusions: The results highlight the significant importance of tumor grade and extent of resection in patient prognosis. The role of adjuvant radiotherapy remains unclear, with most studies suggesting no differences in outcomes, with limitations due to potential confounders.

## 1. Introduction

Spinal astrocytomas (SA) arise from the glial cells of the central nervous system, which serve as supportive cells for neurons. Although intramedullary spinal cord tumors (IMSCTs) are rare [1], SA account for 30–40% of all IMSCTs [2]. In the US, approximately 136 SA are diagnosed each year [3]. Low-grade astrocytomas (WHO 1 and WHO 2) account for 75–80% of all SA, while high-grade SA (WHO 3 and WHO 4) present with poorer outcomes due to their aggressive nature [4]. Despite advancements in treatment modality, managing SA remains challenging. Low-grade SA can be managed through observation, but surgery remains the mainstay for high-grade SA, as recommended by the NCCN guidelines, despite its significant morbidity and the risk of incomplete resection [5].

To enhance treatment modalities strategies for SA, it is crucial to investigate the factors contributing to recurrences. Unlike their well-studied intracranial counterparts, the scarcity of SA limits the availability of large-scale studies, making the current treatment modality largely non-empirical. For example, the role of radiotherapy in SA is unclear as it is primarily used in subtotal resection cases, where most patients receive radiotherapy, making comparisons with a non-radiotherapy group difficult.

The aim of this study was to describe the current standard of care for different grades of SA and identify possible predictors of recurrence in an effort to improve outcomes.

## 2. Materials and Methods

This meta-analysis and systematic review were conducted following the Preferred Reporting Items for Systematic Reviews and Meta-Analyses (PRISMA) guidelines [6]. The protocol applied is similar to previously published works (registration ID: CRD42022330809).

### 2.1. Eligibility Criteria

Peer-reviewed human studies on spinal astrocytomas were included, only when they discussed recurrence. Case reports, reviews, letters, and conference abstracts were excluded.

### 2.2. Databases and Search Strategy

A comprehensive search was conducted across multiple databases, including Ovid MEDLINE(R), Epub Ahead of Print, In-Process & Other Non-Indexed Citations, and Daily, Ovid EMBASE, Ovid Cochrane Central Register of Controlled Trials, Ovid Cochrane Database of Systematic Reviews, and Scopus. The search strategy was designed and conducted by an experienced librarian with input from the study’s principal investigator. Controlled vocabulary and keywords were used to identify studies addressing prognostic factors and treatment outcomes for spinal astrocytomas in humans (Supplementary File S1).

### 2.3. Study Selection

**Figure 1:**
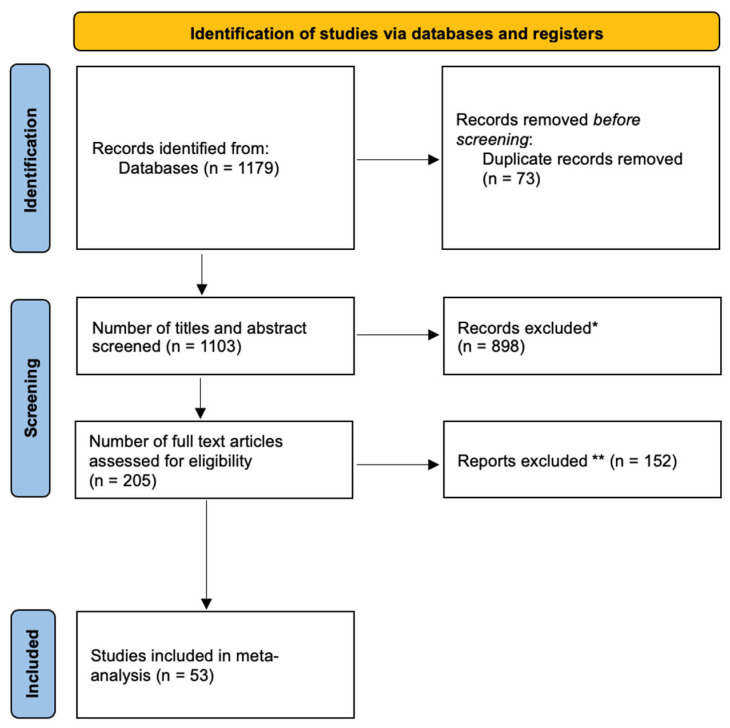
Flowchart of the study inclusion process. * Not spinal astrocytomas. ** Insufficient data on spinal astrocytoma upon full-text review (lack of outcome data or data on mixed tumor subtypes).

Keywords used in the search included “Tumor location”, “histological grade”, “age”, “sex”, and “molecular biomarkers”. The searches yielded 1179 papers, and 73 duplicates were removed. The studies were then uploaded to the systematic review software Rayyan (https://www.rayyan.ai accessed on 15 August 2023) [7] for the reviewing process. One reviewer (H.H.) performed the initial title and abstract screening, excluding 974 studies, leaving 205 studies for full-text screening. Three independent reviewers (H.H., H.M., R.N.) then conducted the final full-text screening and data extraction. The selection process is illustrated in the PRISMA flowchart (Figure 1).

### 2.4. Data Extraction

The following data were extracted: author, year, number of patients, recurrence, overall survival (OS), progression-free survival (PFS), preoperative symptoms, neurological function, the use of IONM, and adjuvant therapies. The extraction was conducted on an electronic spreadsheet.

### 2.5. Statistics

Pooled frequencies and rates were calculated by combining numbers from all included studies. Meta-analyses with fixed or random effect models were performed depending on the calculated heterogeneity among studies. Forest plots comparing recurrences in spinal astrocytomas depending on sex, WHO grading, tumor location, and extent of resection were developed. All statistical analyses were performed using R (version 4.3.2, The R Foundation for Statistical Computing, Vienna, Austria).

## 3. Results

Following the study selection process, a total of 53 studies on 1365 patients specifically addressing recurrence were included (Figure 1). These studies were published between the years of 1982 and 2023 and were largely retrospective in nature. Among these 1365 patients, 45% were males, with a mean age ranging between 5.7 and 43 years, depending on the study. In addition, 693 patients had low-grade tumors (WHO grades 1 and 2), and 412 patients had high-grade tumors (WHO grades 3 and 4), with 598 tumors being cervical, 534 thoracic, and 47 lumbar.

### 3.1. Recurrence

The overall recurrence rate amounted to 41%, with a total of 559 patients experiencing recurrence (Table 1). The median time to recurrence was 18.9 months, with an interquartile range (IQR) of 17.85 months. Stratified data indicated shorter recurrence times for higher-grade tumors. Based on information from 9 studies the 5-year overall survival rate ranged from 19% to 88%. Also, the median survival was reported across 12 studies, varying greatly from 3 months to 102 months.

### 3.2. Neurological Outcome

**Figure 2:**
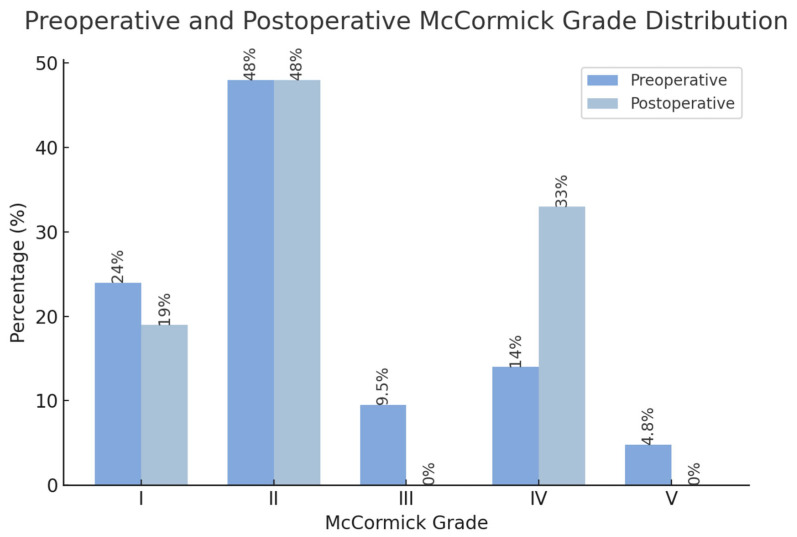
Pre- and postoperative distribution of McCormick grades.

A total of 12 studies provided information on neurological function using the modified McCormick scale, and 6 studies specifically compared changes in modified McCormick grades pre- and postoperatively. A total of 21 patients were described in our set of studies. Preoperatively, 24% were mMC grade I, 48% grade II, 9.5% grade III, 14% grade IV, and 4.8% grade V. Postoperative assessments revealed a shift in this distribution: a decrease to 19% for grade I, no change in grade II, no grade III, an increase to 33% in grade IV, and no grade V (Figure 2).

### 3.3. Meta-Analysis

**Figure 3:**
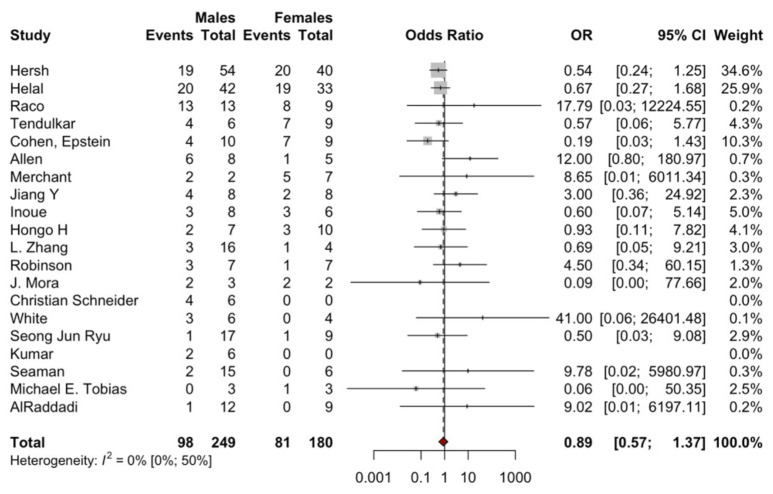
Forest plot comparing recurrences (events) in patients with male vs. female sex. For detailed study references, see File S2.

**Figure 4:**
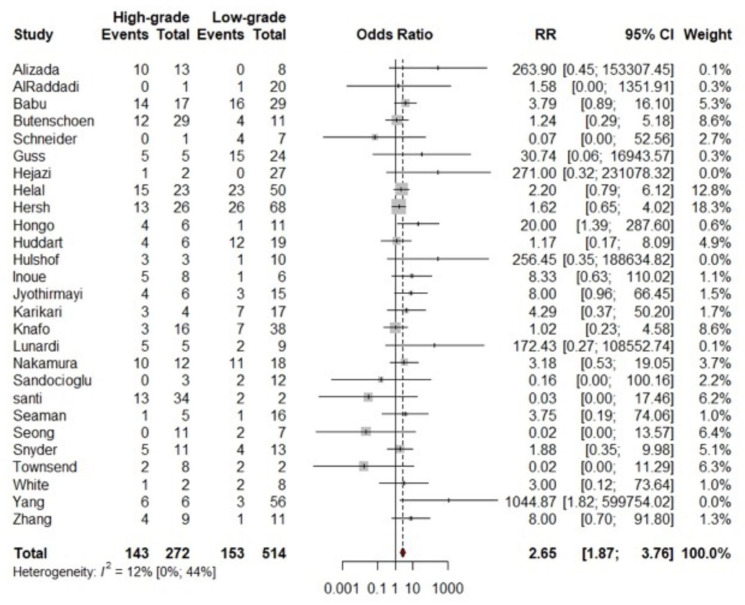
Forest plot comparing recurrences (events) in patients with low vs. high grade SA. For detailed study references, see File S2.

**Figure 5:**
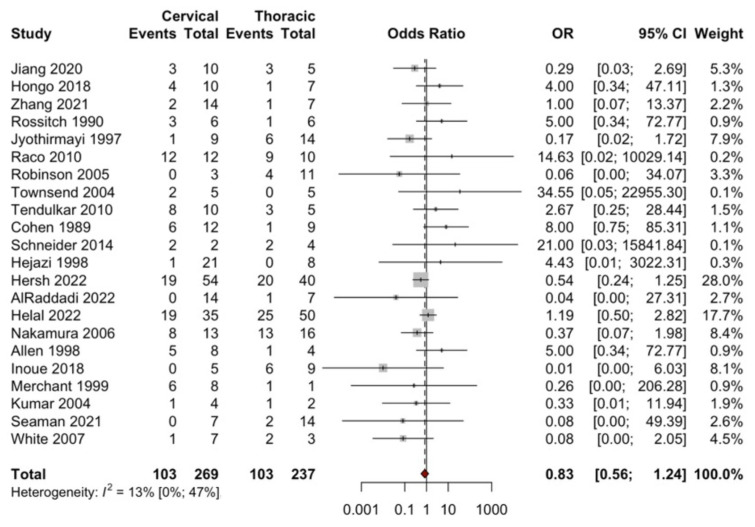
Forest plot comparing recurrences (events) in patients with cervical vs. thoracic SA. For detailed study references, see File S2.

**Figure 6:**
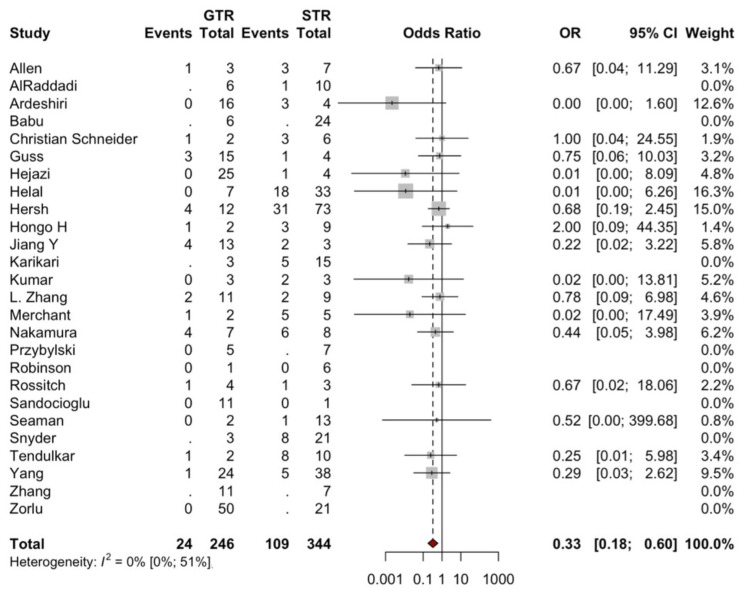
Forest plot comparing recurrences (events) in patients following GTR vs. STR. For detailed study references, see File S2.

In total, 53 studies were used to analyze the factors influencing recurrence in SA. An analysis of 21 studies (Figure 3) showed that male sex was not significantly associated with higher odds of recurrence (OR = 0.89; 95% CI: 0.57, 1.37; *p* = 0.5848). Assessment of 26 studies (Figure 4) showed that high-grade WHO tumors were significantly associated with higher odds of recurrence (OR = 2.65; 95% CI: 1.87, 3.76; *p* = 0.001). Findings from 22 studies (Figure 5) indicated that tumor location was not significantly associated with higher odds of recurrence (OR = 0.83; 95% CI: 0.56, 1.24; *p* = 0.3693). Based on 26 studies (Figure 6), GTR was significantly associated with lower odds of recurrence compared to STR (OR = 0.33; 95% CI: 0.18, 0.60; *p* = 0.0003).

## 4. Discussion

The aim of this study was to describe surgical outcomes and identify prognostic factors in SA, focusing on recurrence factors rather than solely survival rates as the primary outcome. By compiling existing evidence and providing an overview of SA management, this study addresses gaps between recurrence rates and survival outcomes [4].

### 4.1. WHO Grading

As expected, a significant difference in recurrence rates was observed between high-grade and low-grade SA (RR: 2.65, *p* = 0.001). This aligns with previous research, such as the study by Milano et al. [8] and Epstein et al. [9], which similarly identified the WHO grade as the most important predictor of survival in patients with primary SA. Another study reported a median of 18.7 months survival from the time of surgery in patients with high-grade astrocytomas [10]. This meta-analysis demonstrates a significantly higher rate of recurrences in high-grade spinal astrocytomas, which likely contributes to the lower survival rates associated with this tumor.

### 4.2. Extent of Resection

Surgical resection is currently the gold standard in high-grade SA [10]. The results of this meta-analysis are consistent with the current recommendation. Notably, our meta-analysis showed that STR was associated with a threefold increase in recurrences compared to a gross-total resection (GTR). The findings suggest that GTR should be the primary treatment strategy in SA when possible. This is also consistently highlighted in the included literature. For instance, the retrospective study by McGirt et al., involving 35 subjects with SA, demonstrated that patients undergoing STR were associated with a significant decline in overall survival compared to GTR (38% vs. 78% survival at 4 years, *p* = 0.028) [11]. Similarly, another retrospective review of 89 patients with high-grade SA showed the superiority of GTR in survival outcomes compared to STR [12]. Nonetheless, the series of included patients showed an immediate postoperative deterioration in McCormick grades, highlighting the inherent challenge in balancing the extent of tumor resection with the preservation of neurological function. However, clinical experience suggests that many patients experience neurological recovery to certain extents within approximately the first few months post-surgery [13]. This recovery phase indicates that the immediate postoperative McCormick grade should not be a sole determinant of long-term outcomes, emphasizing the need for patience in evaluating surgical success in spinal astrocytoma resections. Importantly, 8 studies discussed the use and benefits of intraoperative neurophysiological monitoring (IONM), highlighting its crucial role in enabling a more radical and safer resection. However, none of the studies provided any empirical evidence supporting its use.

### 4.3. Tumor Location and Tumor Extension

The meta-analysis found no significant difference in recurrence based on tumor location (*p* = 0.369). It is worth mentioning that previous analysis concerning survival had shown the opposite results. The study of Nakamura et al., involving 30 SA patients, suggested better survival with a thoracic as opposed to cervical tumor, for both low-grade (*p* = 0.025) and high-grade astrocytomas (*p* = 0.0126) [14]. Nakamura hypothesized that, compared to cervical SA, a thoracic tumor would take longer to reach the upper cervical cord and cause respiratory failure, which is considered to be the leading cause of death in patients with SA. Nonetheless, in the context of recurrence, most studies did not find tumor location to be a significant predictor of outcomes.

Unfortunately, the analysis of tumor extension was hindered by inconsistent and heterogeneous reporting across the included studies. This variability prevented a pooled analysis in that regard, highlighting the need for standardized reporting in future research.

### 4.4. Sex Differences

The meta-analysis involving 429 patients revealed no significant difference in recurrences based on sex (*p* = 0.585). Previous evidence on the topic revealed inconsistent findings. Wong et al. reported a better survival in male patients compared to female patients (HR = 0.50, 95% CI: 0.29–0.86, *p* = 0.01) in a SEER database study [15]. Some other studies also found a difference in survival between male and female patients favoring males [16,17]. However, Garces-Ambrossi et al. reported no association between sex and overall survival in primary spinal tumors [18].

### 4.5. Radiotherapy

The data did not allow for causal conclusions regarding the effectiveness of radiotherapy as an adjuvant therapy, with the extent of resection consistently acting as a confounding variable. This limitation is common across most of the included studies. Since radiotherapy is most often used in partial resection cases, fair comparison groups are typically lacking. While most studies agree that RT should still be used in cases of partial resection, 10 studies concluded that it did not improve outcomes. For example, Persson et al. [13] found no improvement in outcomes with radiotherapy in partially resected SA in a cohort of 5 patients. Similarly, Nakamura et al. [14] observed no difference in outcomes with radiation in either the low-grade or high-grade group. Bouffet et al. also reported no difference in outcomes in a pediatric cohort of 37 patients with SA [19]. Standardized reporting on radiotherapy dose and applied protocols in future studies is essential to better assess its impact.

### 4.6. Chemotherapy

A total of 18 studies included chemotherapy as a treatment modality with a wide variability, often combined to radiotherapy. The significant heterogeneity in the agents employed and lack of standardization in its use prevent our study from drawing conclusions on the effectiveness of chemotherapy, underscoring the need of further research. Hersh et al. found that chemotherapy was associated with decreased overall survival in his cohort of 54 patient, suggesting that this probably reflects the use of this treatment modality in patients with more aggressive tumors [20].

### 4.7. Survival

A review of 9 studies found that 5-year overall survival ranged from 19% to 88% [14,19,21,22,23,24,25,26,27]. Also, the median survival was reported across 12 studies [26,27,28,29,30,31,32,33,34,35,36,37], varying greatly from 3 months to 102 months, likely due to inherent differences in patient populations and characteristics. Merchant et al. [25] observed that patients experiencing diffuse recurrences had a significantly shorter median overall survival, approximately 10 months, when compared to patients that only recurred locally. Another study from Desousa et al. did not show improved survival with radiotherapy, which may be due to a bias stemming from its use in patients with more advanced or aggressive diseases [26]. Xiao et al. explored the molecular characteristics of spinal astrocytomas but found no correlation between molecular characteristics and overall survival [27]. However, it is important to note that their study primarily involved low-grade tumors, which have a better prognosis, as evidenced by an 83% 5-year survival rate in their cohort. McGirt demonstrated the importance of the surgical approach, with decreased survival in patients undergoing subtotal resection against radical resection in SA (38% vs. 78%) at 4 years. This meta-analysis could not conclusively determine the optimal adjuvant therapy due to the lack of data supporting statistical analysis. This highlights the need for further targeted research to refine treatment modalities, particularly for high-grade and diffuse SA.

### 4.8. Molecular Biomarkers

We attempted to extract data on molecular biomarkers; however, the variability in reported markers, the limited available data, and the highly heterogeneous reported outcomes made it difficult to conduct a meaningful analysis. Subsequently, a limitation of this review is the potential undergrading of spinal astrocytomas due to the lack of advanced molecular information in the reviewed articles’ tumor grading. It is now recognized that WHO grade 1 or 2 spinal astrocytomas may harbor H3F3A K27M mutations and/or loss of H3K27me3 staining, which make them more aggressive and correspond to higher-grade tumors. For instance, Tanaka et al. [38], in a recent retrospective study of 25 patients, and Biczok et al. [39] demonstrated these molecular alterations significantly worsen both tumor grading and prognosis. Larger studies are needed to determine the role of these biomarkers in tumor progression and outcome prediction.

### 4.9. Limitations

The principal limitation of this meta-analysis is the inherent retrospective nature of the included studies. This most certainly limits our capacity to draw causal inferences, a common limitation in this type of study due to all the potential confounding variables. Also, most of the included studies lacked certain data points, as well as data could be used in the context of a meta-analysis, which decreased the power of the final analysis. Data on adjuvant therapy and tumor markers were not granular enough to include in the statistical analysis.

A limitation of this study is the inclusion of studies published over a long period of time. Medical progress during this period likely introduced variability and potential bias in the pooled data. However, given the rarity of spinal astrocytomas, this broad inclusion was necessary to gather sufficient data for meaningful analysis.

## 5. Conclusions

The overall rate of recurrence in patients with spinal astrocytomas amounted to over 40%. Using meta-analytic statistics, the current findings from this review corroborate previously established knowledge surrounding SA. Notably, both extent of resection and WHO grading play a crucial role in patient prognosis, as higher-grade tumors and STR are associated with a significantly higher relative risk of recurrences. Additional factors such as tumor location or sex were studied, revealing no significant association with recurrence. The role of adjuvant radiotherapy remains unclear, with most studies suggesting no differences in outcomes, with limitations due to potential confounders. Tumor biomarkers in the context of SA remain an area of interest and warrant further investigations.

## Figures and Tables

**Table 1:** Study characteristics including total number of patients, extent of resection, WHO grade, number of confirmed recurrences, and the use of adjuvant therapies. A comprehensive list of references is provided in Supplementary File S2.

Author	Total Number of Patients	Gross Total Resection	Subtotal Resection	Biopsy Only	WHO Grade 1	WHO Grade 2	WHO Grade 3	WHO Grade 4	Recurrence	Radiotherapy	Chemotherapy	Both Radio and Chemotherapy
Kopelson et al., 1982	14	-	-	-	-	-	-	7	1	13	-	-
Cooper et al., 1985	11	-	-	-	3	5	2	1	3	-	-	-
Naidu et al., 1989	11	-	-	-	-	-	-	-	0	6	-	-
Cohen et al., 1989	19	11	-	-	-	-	9	10	11	-	-	-
Rossitch et al., 1990	12	1	1	2	12	0	0	0	4	7	-	-
Hulshof et al., 1993	13	0	0	6	-	-	1	10	6	13	-	-
Lunardi et al., 1993	14	-	-	-	-	-	-	9	7	5	-	-
Huddart et al., 1993	27	0	-	-	11	8	5	1	16	-	-	-
Mark et al., 1996	3	-	-	-	0	2	0	1	1	-	-	-
Jyothirmayi et al., 1997	23	-	-	-	0	0	0	0	7	23	-	-
Allen et al., 1998	13	1	3	3	0	0	9	4	7	10	13	-
Hejazi et al., 1998	29	0	1	0	-	-	-	-	1	-	-	-
Bouffet et al., 1998	73	-	-	-	28	21	21	3	17	37	-	3
Merchant et al., 1999	9	1	5	1	-	-	-	-	7	9	6	6
Lee et al., 2003	25	-	-	-	8	7	4	6	18	22	-	-
Santi et al., 2003	36	-	-	-	0	2	13	21	15	10	-	7
Kumar et al., 2004	6	0	2	0	-	-	-	-	2	6	-	-
Townsend et al., 2004	10	-	-	-	-	-	-	-	4	2	4	2
Robinson et al., 2005	14	0	0	4	-	-	-	-	4	10	-	-
Sandocioglu et al., 2005	15	0	0	2	-	-	-	12	2	-	-	-
Nakamura et al., 2006	30	4	6	11	7	11	11	1	21	19	-	-
White et al., 2007	10	0	2	1	-	-	-	8	3	-	-	-
Tobias et al., 2008	6	-	-	-	2	4	0	0	1	-	-	-
Yang et al., 2009	62	1	5	0	-	-	-	-	9	39	-	-
Eroes et al., 2010	15	0	2	0	8	7	0	0	2	2	-	-
Tendulkar et al., 2010	15	1	8	2	0	0	8	7	11	15	7	-
Raco et al., 2010	22	2	-	-	0	0	10	12	21	15	-	12
Karikari et al., 2011	21	-	5	-	12	5	3	1	10	-	-	-
Ardeshiri et al., 2013	22	0	3	1	-	-	3	4	3	-	-	-
Guss et al., 2013	29	3	1	-	-	-	-	-	20	12	13	-
Schneider et al., 2014	6	1	3	0	3	2	0	1	4	2	2	-
Babu et al., 2014	46	-	-	-	19	10	9	8	30	26	30	-
Sahu et al., 2015	11	1	0	0	-	-	-	-	1	-	-	-
Ryu et al., 2016	26	2	0	0	-	-	-	-	2	-	-	-
Mora et al., 2018	5	-	-	3	1	3	0	0	4	-	-	-
Inoue et al., 2018	14	0	1	5	-	-	-	-	6	13	9	-
Hongo et al., 2018	17	1	3	1	2	9	5	1	5	4	2	5
Zou et al., 2018	94	-	-	-	20	22	23	25	64	54	53	-
Jiang et al., 2020	16	4	2	0	16	0	0	0	6	0	0	0
Alizada et al., 2020	21	-	-	-	3	5	9	4	10	13	13	-
Zhang et al., 2021	20	2	2	0	-	-	-	11	4	4	3	4
Seaman et al., 2021	21	0	1	-	-	-	-	16	2	13	7	-
Butenschoen et al., 2021	40	-	-	-	0	11	12	17	16	3	-	4
Knafo et al., 2021	54	-	-	-	19	19	11	5	10	16	4	4
AlRaddadi et al., 2022	21	-	1	-	10	10	1	0	1	9	-	-
Snyder et al., 2022	24	-	8	-	7	6	7	4	9	4	5	6
Helal et al., 2022	75	0	18	21	38	12	12	11	39	25	-	25
Hersh et al., 2022	94	4	31	4	40	28	11	15	39	35	36	-

A dash «-» is represented when data were unavailable.

## Data Availability

The original contributions presented in this study are included in the article. Further inquiries can be directed to the corresponding author.

## Supplementary Materials

The following supporting information can be downloaded at: https://www.mdpi.com/article/10.3390/brainsci14121226/s1, File S1: Actual Search Strategies. File S2: Full list of references for studies included in the meta-analysis.
