# Supplementary material for: Determining the Predictors of Recurrence or Regrowth Following Spinal Astrocytoma Resection: A Systematic Review and Meta-Analysis
Source: Brain Sci. 2024 Dec 4;14(12):1226. doi: 10.3390/brainsci14121226 (PMC11726993; doi:10.3390/brainsci14121226)
Supplement: Supplementary file 1 [file brainsci-14-01226-s001.zip › Supplementary File S1.pdf]

Ovid

Database(s): EBM Reviews - Cochrane Central Register of Controlled Trials July 2023, EBM Reviews - Cochrane Database of Systematic Reviews 2005 to August 9, 2023, Embase 1974 to 2023 August 11, Ovid MEDLINE(R) and Epub Ahead of Print, In-Process, In-Data-Review & Other Non-Indexed Citations, Daily and Versions 1946 to August 11, 2023
Search Strategy:

#	Searches	Results
1	exp Spinal Cord Neoplasms/ and exp Astrocytoma/	2389
2	((spine or spinal) adj4 ("astrocytic glioma" or "astrocytic gliomas" or astrocytoma or astrocytomas or astroglioma or astrogliomas or "mixed oligoastrocytoma" or "mixed oligoastrocytomas" or "pleomorphic xanthoastrocytoma" or "pleomorphic xanthoastrocytomas")).ti,ab,kf.	835
3	1 or 2	2724
4	exp Prognosis/	3043277
5	exp Biomarkers, Tumor/	698806
6	su.fs.	4703798
7	exp Neurosurgical Procedures/	527091
8	exp Biopsy/	1227340
9	rt.fs.	603546
10	exp Radiotherapy/	863969
11	exp Chemoradiotherapy/	97514
12	dt.fs.	7309708
13	exp Drug Therapy/	5168651
14	exp Neoplasm Grading/	151594
15	exp Age Factors/	1605918
16	exp Sex Factors/	299696
17	exp Risk Assessment/	1063027
18	(adjuvant or age or anterior or biomarker* or biops* or "BRAF V600E" or cervical or characteristic or characteristics or chemoradiotherap* or Clinicopathological or debulk* or demographic* or dorsal or "dorsal root" or dorsolateral or "drug therap*" or "entry zone" or gender or "gleason score*" or grade or grades or grading or "H3 K27M" or histological or IDH1 or intermediate or "isocitrate dehydrogenase" or "Ki-67" or laminectom* or laminoplast* or location or lumbar or marker or markers or "Modified McCormick Scale" or molecular or mutation* or myelotomy or neurosurg* or Nomogram* or operat* or pharmacotherap* or "posterior midline" or predict* or procedure* or prognosis or prognostic or R132H or radiotherap* or resect* or "risk assessment*" or "risk stratification*" or "segment length" or sex or stage or stages or staging or surg* or temozolomide or "TERT promoter" or thoracic or "WHO classification*" or wildtype or "World Health Organization classification*").ti,ab,kf.	32882654
19	or/4-18	40914961

20	3 and 19	2388
21	(case adj3 report).mp,pt.	3587868
22	20 not 21	1702
23	limit 22 to english language [Limit not valid in CDSR; records were retained]	1518
24	limit 22 to no language specified [Limit not valid in CDSR; records were retained]	1
25	23 or 24	1519
26	limit 25 to (editorial or erratum or note or addresses or autobiography or bibliography or biography or blogs or comment or dictionary or directory or interactive tutorial or interview or lectures or legal cases or legislation or news or newspaper article or overall or patient education handout or periodical index or portraits or published erratum or webcasts) [Limit not valid in CCTR,CDSR,Embase,Ovid MEDLINE(R); records were retained]	42
27	25 not 26	1477
28	(exp animals/ or exp nonhuman/) not (exp humans/ or exp patient/)	12249583
29	((alpaca or alpacas or amphibian or amphibians or animal or animals or antelope or armadillo or armadillos or avian or baboon or baboons or beagle or beagles or bee or bees or bird or birds or bison or bovine or buffalo or buffaloes or buffalos or "c elegans" or "Caenorhabditis elegans" or camel or camels or canine or canines or carp or cats or cattle or chick or chicken or chickens or chicks or chimp or chimpanze or chimpanzees or chimps or cow or cows or "D melanogaster" or "dairy calf" or "dairy calves" or deer or dog or dogs or donkey or donkeys or drosophila or "Drosophila melanogaster" or duck or duckling or ducklings or ducks or equid or equids or equine or equines or feline or felines or ferret or ferrets or finch or finches or fish or flatworm or flatworms or fox or foxes or frog or frogs or "fruit flies" or "fruit fly" or "G mellonella" or "Galleria mellonella" or geese or gerbil or gerbils or goat or goats or goose or gorilla or gorillas or hamster or hamsters or hare or hares or heifer or heifers or horse or horses or insect or insects or jellyfish or kangaroo or kangaroos or kitten or kittens or lagomorph or lagomorphs or lamb or lambs or lemur or lemurs or llama or llamas or macaque or macaques or macaw or macaws or marmoset or marmosets or mice or minipig or minipigs or mink or minks or monkey or monkeys or mouse or mule or mules or nematode or nematodes or octopus or octopuses or orangutan or "orang-utan" or orangutans or "orang-utans" or ostrich or ostriches or oxen or parrot or parrots or pig or pigeon or pigeons or piglet or piglets or pigs or porcine or primate or primates or quail or rabbit or rabbits or rat or rats or reptile or reptiles or rodent or rodents or ruminant or ruminants or salmon or sheep or shrimp or slug or slugs or swine or tamarin or tamarins or toad or toads or trout or urchin or urchins or vole or voles or waxworm or waxworms or wildlife or worm or worms or xenopus or "zebra fish" or zebrafish) not (human or humans or patient or patients)).ti,ab,hw,kf.	10483050
30	27 not (28 or 29)	1437
31	remove duplicates from 30	1178

Scopus

1 TITLE-ABS-KEY(((spine or spinal) W/4 ("astrocytic glioma" OR "astrocytic gliomas" OR astrocytoma OR astrocytomas OR astroglioma OR astrogliomas OR "mixed oligoastrocytoma" OR "mixed oligoastrocytomas" OR "pleomorphic xanthoastrocytoma" OR "pleomorphic xanthoastrocytomas")))

2 TITLE-ABS-KEY(adjuvant OR age OR anterior OR biomarker* OR biops* OR "BRAF V600E" OR cervical OR characteristic OR characteristics OR chemoradiotherap* OR Clinicopathological OR debulk* OR demographic* OR dorsal OR "dorsal root" OR dorsolateral OR "drug therap*" OR "entry zone" OR gender OR "gleason score*" OR grade OR grades OR grading OR "H3 K27M" OR histological OR IDH1 OR intermediate OR "isocitrate dehydrogenase" OR "Ki-67" OR laminectom* OR laminoplast* OR location OR lumbar OR marker OR markers OR "Modified McCormick Scale" OR molecular OR mutation* OR myelotomy OR neurosurg* OR Nomogram* OR operat* OR pharmacotherap* OR "posterior midline" OR predict* OR procedure* OR prognosis OR prognostic OR R132H OR radiotherap* OR resect* OR "risk assessment*" OR "risk stratification*" OR "segment length" OR sex OR stage OR stages OR staging OR surg* OR temozolomide OR "TERT promoter" OR thoracic OR "WHO classification*" OR wildtype OR "World Health Organization classification*")

3 LANGUAGE(english)

4 1 and 2 and 3

5 TITLE-ABS-KEY(case W/3 report)

6 4 and not 5

7 DOCTYPE(ed) OR DOCTYPE(bk) OR DOCTYPE(er) OR DOCTYPE(no) OR DOCTYPE(sh)

8 6 and not 7

9 TITLE-ABS-KEY((alpaca OR alpacas OR amphibian OR amphibians OR animal OR animals OR antelope OR armadillo OR armadillos OR avian OR baboon OR baboons OR beagle OR beagles OR bee OR bees OR bird OR birds OR bison OR bovine OR buffalo OR buffaloes OR buffalos OR "c elegans" OR "Caenorhabditis elegans" OR camel OR camels OR canine OR canines OR carp OR cats OR cattle OR chick OR chicken OR chickens OR chicks OR chimp OR chimpanze OR chimpanzees OR chimps OR cow OR cows OR "D melanogaster" OR "dairy calf" OR "dairy calves" OR deer OR dog OR dogs OR donkey OR donkeys OR drosophila OR "Drosophila melanogaster" OR duck OR duckling OR ducklings OR ducks OR equid OR equids OR equine OR equines OR feline OR felines OR ferret OR ferrets OR finch OR finches OR fish OR flatworm OR flatworms OR fox OR foxes OR frog OR frogs OR "fruit flies" OR "fruit fly" OR "G mellonella" OR "Galleria mellonella" OR geese OR gerbil OR gerbils OR goat OR goats OR goose OR gorilla OR gorillas OR hamster OR hamsters OR hare OR hares OR heifer OR heifers OR horse OR horses OR insect OR insects OR jellyfish OR kangaroo OR kangaroos OR kitten OR kittens OR lagomorph OR lagomorphs OR lamb OR lambs OR llama OR llamas OR macaque OR macaques OR macaw OR macaws OR marmoset OR marmosets OR mice OR minipig OR minipigs OR mink OR minks OR monkey OR monkeys OR mouse OR mule OR mules OR nematode OR nematodes OR octopus OR octopuses OR orangutan OR "orang-utan" OR orangutans OR "orang-utans" OR oxen OR parrot OR parrots OR pig OR pigeon OR pigeons OR piglet OR piglets OR pigs OR porcine OR primate OR primates OR quail OR rabbit OR rabbits OR rat OR rats OR reptile OR reptiles OR rodent OR rodents OR ruminant OR ruminants OR salmon OR sheep OR shrimp OR slug OR slugs OR swine OR tamarin OR tamarins OR toad OR toads OR trout OR urchin OR urchins OR vole OR voles OR waxworm OR waxworms OR worm OR worms OR xenopus OR "zebra fish" OR zebrafish) AND NOT (human OR humans or patient or patients))

10 8 and not 9

11	INDEX(embase) OR INDEX(medline) OR PMID(0* OR 1* OR 2* OR 3* OR 4* OR 5* OR 6* OR 7* OR 8* OR 9*)
12	10 and not 11
