# Supplementary material for: Determining the Predictors of Recurrence or Regrowth Following Spinal Astrocytoma Resection: A Systematic Review and Meta-Analysis
Source: Brain Sci. 2024 Dec 4;14(12):1226. doi: 10.3390/brainsci14121226 (PMC11726993; doi:10.3390/brainsci14121226)
Supplement: Supplementary file 1 [file brainsci-14-01226-s001.zip › Supplementary File S2.pdf]

Authors and Title
Kopelson G, Linggood RM. Intramedullary spinal cord astrocytoma versus glioblastoma: the prognostic importance of histologic grade. Cancer [Internet]. 1982 Aug 15;50(4):732–5. Available from: http://dx.doi.org/10.1002/1097-0142(19820815)50:4<732::aid-cncr2820500418>3.0.co;2-0
Cooper PR, Epstein F. Radical resection of intramedullary spinal cord tumors in adults. Recent experience in 29 patients. J Neurosurg [Internet]. 1985 Oct;63(4):492–9. Available from: http://dx.doi.org/10.3171/jns.1985.63.4.0492
Naidu MR, Dinakar I. Intramedullary mass lesions of the spinal cord. Clin Neurol Neurosurg [Internet]. 1989;91(2):135–8. Available from: http://dx.doi.org/10.1016/s0303-8467(89)80034-4
Cohen AR, Wisoff JH, Allen JC, Epstein F. Malignant astrocytomas of the spinal cord. J Neurosurg [Internet]. 1989 Jan;70(1):50–4. Available from: http://dx.doi.org/10.3171/jns.1989.70.1.0050
Rossitch E Jr, Zeidman SM, Burger PC, Curnes JT, Harsh C, Anscher M, et al. Clinical and pathological analysis of spinal cord astrocytomas in children. Neurosurgery [Internet]. 1990 Aug 1;27(2):193–6. Available from: http://dx.doi.org/10.1227/00006123-199008000-00003
Hulshof MCCM, Menten J, Dito JJ, Dreissen JJR, van den Bergh R, González González D. Treatment results in primary intraspinal gliomas. Radiother Oncol [Internet]. 1993 Dec;29(3):294–300. Available from: http://dx.doi.org/10.1016/0167-8140(93)90147-z
Lunardi P, Cervoni L, Maleci A, Fortuna A. Isolated haemangioblastoma of spinal cord: report of 18 cases and a review of the literature. Acta Neurochir (Wien) [Internet]. 1993;122(3–4):236–9. Available from: http://dx.doi.org/10.1007/bf01405535
Huddart R, Traish D, Ashley S, Moore A, Brada M. Management of spinal astrocytoma with conservative surgery and radiotherapy. Br J Neurosurg [Internet]. 1993;7(5):473–81. Available from: http://dx.doi.org/10.3109/02688699308995069
Lee M, Rezai AR, Freed D, Epstein FJ. Intramedullary spinal cord tumors in neurofibromatosis. Neurosurgery [Internet]. 1996 Jan;38(1):32–7. Available from: http://dx.doi.org/10.1097/00006123-199601000-00009
Jyothirmayi R, Madhavan J, Nair MK, Rajan B. Conservative surgery and radiotherapy in the treatment of spinal cord astrocytoma. J Neurooncol [Internet]. 1997 Jul;33(3):205–11. Available from: http://dx.doi.org/10.1023/a:1005758313700

Allen JC, Aviner S, Yates AJ, Boyett JM, Cherlow JM, Turski PA, et al. Treatment of high-grade spinal cord astrocytoma of childhood with “8-in-1” chemotherapy and radiotherapy: a pilot study of CCG-945. Children’s Cancer Group. J Neurosurg [Internet]. 1998 Feb;88(2):215–20. Available from: http://dx.doi.org/10.3171/jns.1998.88.2.0215

Hejazi N, Hassler W. Microsurgical treatment of intramedullary spinal cord tumors: Results of 80 patients and review of the relevant literature. Neurosurgery [Internet]. 1998 Sep;43(3):675–675. Available from: http://dx.doi.org/10.1097/00006123-199809000-00194

Bouffet E, Pierre-Kahn A, Marchal JC, Jouvet A, Kalifa C, Choux M, et al. Prognostic factors in pediatric spinal cord astrocytoma. Cancer [Internet]. 1998 Dec 1;83(11):2391–9. Available from: http://dx.doi.org/10.1002/(sici)1097-0142(19981201)83:11<2391::aid-cncr20>3.0.co;2-0

Merchant TE, Nguyen D, Thompson SJ, Reardon DA, Kun LE, Sanford RA. High-grade pediatric spinal cord tumors. Pediatr Neurosurg [Internet]. 1999 Jan;30(1):1–5. Available from: http://dx.doi.org/10.1159/000028751

Lee HK, Chang EL, Fuller GN, Aldape KD, Atkinson GJ, Levy LB, et al. The prognostic value of neurologic function in astrocytic spinal cord glioma. Neuro Oncol [Internet]. 2003 Jul;5(3):208–13. Available from: http://dx.doi.org/10.1215/S1152851702000595

Santi M, Mena H, Wong K, Koeller K, Olsen C, Rushing EJ. Spinal cord malignant astrocytomas. Clinicopathologic features in 36 cases. Cancer [Internet]. 2003 Aug 1;98(3):554–61. Available from: http://dx.doi.org/10.1002/cncr.11514

Kumar R, Singh V. Intramedullary mass lesion of the spinal cord in children of a developing milieu. Pediatr Neurosurg [Internet]. 2004 Jan;40(1):16–22. Available from: http://dx.doi.org/10.1159/000076572

Townsend N, Handler M, Fleitz J, Foreman N. Intramedullary spinal cord astrocytomas in children. Pediatr Blood Cancer [Internet]. 2004 Nov;43(6):629–32. Available from: http://dx.doi.org/10.1002/pbc.20082

Robinson CG, Prayson RA, Hahn JF, Kalfas IH, Whitfield MD, Lee S-Y, et al. Long-term survival and functional status of patients with low-grade astrocytoma of spinal cord. Int J Radiat Oncol Biol Phys [Internet]. 2005 Sep 1;63(1):91–100. Available from: http://dx.doi.org/10.1016/j.ijrobp.2005.01.009

Sandalcioglu IE, Gasser T, Asgari S, Lazorisak A, Engelhorn T, Egelhof T, et al. Functional outcome after surgical treatment of intramedullary spinal cord tumors: experience with 78 patients. Spinal Cord [Internet]. 2005 Jan;43(1):34–41. Available from: http://dx.doi.org/10.1038/sj.sc.3101668

Nakamura M, Chiba K, Ishii K, Ogawa Y, Takaishi H, Matsumoto M, et al. Surgical outcomes of spinal cord astrocytomas. Spinal Cord [Internet]. 2006 Dec;44(12):740–5. Available from: http://dx.doi.org/10.1038/sj.sc.3101932

White JB, Miller GM, Layton KF, Krauss WE. Nonenhancing tumors of the spinal cord. J Neurosurg Spine [Internet]. 2007 Oct;7(4):403–7. Available from: http://dx.doi.org/10.3171/SPI-07/09/10/403

Tobias ME, McGirt MJ, Chaichana KL, Goldstein IM, Kothbauer KF, Epstein F, et al. Surgical management of long intramedullary spinal cord tumors. Childs Nerv Syst [Internet]. 2008 Feb;24(2):219–23. Available from: http://dx.doi.org/10.1007/s00381-007-0405-7

Yang S, Yang X, Hong G. Surgical treatment of one hundred seventy-four intramedullary spinal cord tumors. Spine (Phila Pa 1976) [Internet]. 2009 Nov 15;34(24):2705–10. Available from: http://dx.doi.org/10.1097/BRS.0b013e3181b43484

Eroes CA, Zausinger S, Kreth F-W, Goldbrunner R, Tonn J-C. Intramedullary low grade astrocytoma and ependymoma. Surgical results and predicting factors for clinical outcome. Acta Neurochir (Wien) [Internet]. 2010 Apr;152(4):611–8. Available from: http://dx.doi.org/10.1007/s00701-009-0577-x

Tendulkar RD, Pai Panandiker AS, Wu S, Kun LE, Broniscer A, Sanford RA, et al. Irradiation of pediatric high-grade spinal cord tumors. Int J Radiat Oncol Biol Phys [Internet]. 2010 Dec 1;78(5):1451–6. Available from: http://dx.doi.org/10.1016/j.ijrobp.2009.09.071

Raco A, Piccirilli M, Landi A, Lenzi J, Delfini R, Cantore G. High-grade intramedullary astrocytomas: 30 years' experience at the Neurosurgery Department of the University of Rome "Sapienza." J Neurosurg Spine [Internet]. 2010 Feb;12(2):144–53. Available from: http://dx.doi.org/10.3171/2009.6.SPINE08910

Karikari IO, Nimjee SM, Hodges TR, Cutrell E, Hughes BD, Powers CJ, et al. Impact of tumor histology on resectability and neurological outcome in primary intramedullary spinal cord tumors: a single-center experience with 102 patients. Neurosurgery [Internet]. 2011 Jan;68(1):188–97; discussion 197. Available from: http://dx.doi.org/10.1227/NEU.0b013e3181fe3794

Ardeshiri A, Chen B, Hütter B-O, Oezkan N, Wanke I, Sure U, et al. Intramedullary spinal cord astrocytomas: the influence of localization and tumor extension on resectability and functional outcome. Acta Neurochir (Wien) [Internet]. 2013 Jul;155(7):1203–7. Available from: http://dx.doi.org/10.1007/s00701-013-1762-5

Guss ZD, Moningi S, Jallo GI, Cohen KJ, Wharam MD, Terezakis SA. Management of pediatric spinal cord astrocytomas: outcomes with adjuvant radiation. Int J Radiat Oncol Biol Phys [Internet]. 2013 Apr 1;85(5):1307–11. Available from: http://dx.doi.org/10.1016/j.ijrobp.2012.11.022

Schneider C, Hidalgo ET, Schmitt-Mechelke T, Kothbauer KF. Quality of life after surgical treatment of primary intramedullary spinal cord tumors in children. J Neurosurg Pediatr [Internet]. 2014 Feb;13(2):170–7. Available from: http://dx.doi.org/10.3171/2013.11.peds13346

Babu R, Karikari IO, Owens TR, Bagley CA. Spinal cord astrocytomas: a modern 20-year experience at a single institution. Spine (Phila Pa 1976) [Internet]. 2014 Apr 1;39(7):533–40. Available from: http://dx.doi.org/10.1097/BRS.0000000000000190

Sahu RK, Das KK, Bhaisora KS, Singh AK, Mehrotra A, Srivastava AK, et al. Pediatric intramedullary spinal cord lesions: Pathological spectrum and outcome of surgery. J Pediatr Neurosci [Internet]. 2015 Jul;10(3):214–21. Available from: http://dx.doi.org/10.4103/1817-1745.165660

Ryu SJ, Kim JY, Kim KH, Park JY, Kuh SU, Chin DK, et al. A retrospective observational study on the treatment outcomes of 26 patients with spinal cord astrocytoma including two cases of malignant transformation. Eur Spine J [Internet]. 2016 Dec;25(12):4067–79. Available from: http://dx.doi.org/10.1007/s00586-016-4475-7

Mora J, Perez-Jaume S, Cruz O. Treatment of childhood astrocytomas with irinotecan and cisplatin. Clin Transl Oncol [Internet]. 2018 Apr;20(4):500–7. Available from: http://dx.doi.org/10.1007/s12094-017-1741-z

Inoue T, Endo T, Nakamura T, Shibahara I, Endo H, Tominaga T. Expression of CD133 as a putative prognostic biomarker to predict intracranial dissemination of primary spinal cord astrocytoma. World Neurosurg [Internet]. 2018 Feb;110:e715–26. Available from: http://dx.doi.org/10.1016/j.wneu.2017.11.089

Hongo H, Takai K, Komori T, Taniguchi M. Intramedullary spinal cord ependymoma and astrocytoma: intraoperative frozen-section diagnosis, extent of resection, and outcomes. J Neurosurg Spine [Internet]. 2019 Jan 1;30(1):133–9. Available from: http://dx.doi.org/10.3171/2018.7.SPINE18230

Zou Y, Sun J, Zhou Y, Bai HX, Huang X, Babu R, et al. Prognostic factors and treatment of spinal astrocytomas: A multi-institutional cohort analysis. Spine (Phila Pa 1976) [Internet]. 2018 May 15;43(10):E565–73. Available from: http://dx.doi.org/10.1097/BRS.0000000000002485

Jiang Y, Lv L, Yin S, Zhou P, Jiang S. Primary spinal pilocytic astrocytoma: clinical study with long-term follow-up in 16 patients and a literature review. Neurosurg Rev [Internet]. 2020 Apr;43(2):719–27. Available from: http://dx.doi.org/10.1007/s10143-019-01109-0

Alizada O, Kemerdere R, Ulu MO, Akgun MY, Isler C, Kizilkilic O, et al. Surgical management of spinal intramedullary tumors: Ten-year experience in a single institution. J Clin Neurosci [Internet]. 2020 Mar;73:201–8. Available from: http://dx.doi.org/10.1016/j.jocn.2019.12.054

Zhang L, Li T, Qiao G, Yang W, Shang A, Yu X. Clinical characteristics and long-term surgical outcomes of spinal pilocytic astrocytoma: a report of twenty cases. Acta Neurochir (Wien) [Internet]. 2021 Nov;163(11):3005–13. Available from: http://dx.doi.org/10.1007/s00701-020-04606-0

Seaman SC, Bathla G, Park BJ, Woodroffe RW, Smith M, Menezes AH, et al. MRI characteristics and resectability in spinal cord glioma. Clin Neurol Neurosurg [Internet]. 2021 Jan;200(106321):106321. Available from: http://dx.doi.org/10.1016/j.clineuro.2020.106321

Butenschoen VM, Hubertus V, Janssen IK, Onken J, Wipplinger C, Mende KC, et al. Surgical treatment and neurological outcome of infiltrating intramedullary astrocytoma WHO II-IV: a multicenter retrospective case series. J Neurooncol [Internet]. 2021 Jan;151(2):181–91. Available from: http://dx.doi.org/10.1007/s11060-020-03647-w

Knafo S, Aghakhani N, David P, Parker F. Management of intramedullary spinal cord tumors: A single-center experience of 247 patients. Rev Neurol (Paris) [Internet]. 2021 May;177(5):508–14. Available from: http://dx.doi.org/10.1016/j.neurol.2020.07.014

AlRaddadi KK, Farrash F, Baeesa S, Alkhani AM. Primary spinal intramedullary astrocytomas; long-term outcomes and literature review. Interdiscip Neurosurg [Internet]. 2022 Mar;27(101401):101401. Available from: http://dx.doi.org/10.1016/j.inat.2021.101401

Snyder MH, Yu-Der Wang A, Ampie L, Sarathy D, Chatrath A, Asthagiri AR, et al. Primary spinal cord astrocytomas: Two-center clinical experience of low- and high-grade lesions. World Neurosurg [Internet]. 2022 Nov;167:e1006–16. Available from: http://dx.doi.org/10.1016/j.wneu.2022.08.130

Helal A, Alvi MA, Everson M, Mikula AL, Cohen Cohen S, Bydon M, et al. Prognostic factors independently associated with improved progression-free survival after surgical resection in patients with spinal cord astrocytomas: An institutional case series. Oper Neurosurg (Hagerstown) [Internet]. 2022 Mar 1;22(3):106–14. Available from: http://dx.doi.org/10.1227/ONS.0000000000000084

Hersh AM, Antar A, Pennington Z, Aygun N, Patel J, Goldsborough E 3rd, et al. Predictors of survival and time to progression following operative management of intramedullary spinal cord astrocytomas. J Neurooncol [Internet]. 2022 May;158(1):117–27. Available from: http://dx.doi.org/10.1007/s11060-022-04017-4
